# The Mutual Influence of Thermal Contact Conductivity and Convective Cooling on the Temperature Field in a Tribosystem with a Functionally Graded Strip

**DOI:** 10.3390/ma16227126

**Published:** 2023-11-10

**Authors:** Aleksander Yevtushenko, Katarzyna Topczewska, Przemysław Zamojski

**Affiliations:** Faculty of Mechanical Engineering, Bialystok University of Technology (BUT), 45C Wiejska Street, 15-351 Bialystok, Poland; a.yevtushenko@pb.edu.pl (A.Y.); p.zamojski@pb.edu.pl (P.Z.)

**Keywords:** functionally graded material, frictional heating, temperature, thermal contact conductivity, convective cooling

## Abstract

An analytical model to find the temperature field that has been developed for friction systems consists of a strip and semi-space. The strip is made of a two-component functionally graded material (FGM) with an exponentially changing coefficient of thermal conductivity. In contrast, the material of the semi-space is homogeneous. An appropriate boundary-value problem of heat conduction with constant specific friction power was formulated and solved using the Laplace integral transform method. The model takes into consideration the imperfect thermal friction contact between the strip and the semi-space, and also the convective cooling on the exposed surface of the strip. The appropriate asymptotic solutions to this problem for low and high values of Fourier number were obtained. It is shown how the determined exact solution can be generalized using Duhamel’s formula for the case of a linearly reduction in time-specific friction power (a braking process with constant deceleration). Numerical analysis for selected materials of the friction pair was carried out in terms of examining the mutual impact on the temperature of the two Biot numbers, characterizing the intensity of the thermal contact conductivity and convective heat exchange on the exposed surface of the strip. The obtained results can be used to predict the temperature of friction systems containing elements made of FGM. In particular, such systems include modern disc braking systems.

## 1. Introduction

In order to model the frictional heating process at the interface of elements in sliding contact, the thermal problems of friction are formulated in the form of classical heat conduction equations with the appropriate boundary and initial conditions. Solutions of such problems result in temperature distributions in both components of the friction pair. One of the various approaches used during the formulation of the heat conduction problems is the separated bodies concept [1,2], which considers the sliding elements of the friction pair to be uncoupled. Naturally, this simplification forces the introduction of a virtual heat source in order to take into account relevant thermal effects associated with frictional contact interactions. The heat flux absorbed on the friction surface by each component is then established using a suitable heat partition ratio [3,4]. This coefficient is mostly introduced to the models a priori, which might be determined based on experimental studies or analytical solutions [4,5,6]. 

Unlike the separation approach, the formulation for the thermal friction problems in accordance with the second variant assumes the presence of two bodies in contact. In coupled problems, it is necessary to formulate the boundary conditions at the interface between bodies [7]. Most often, it is assumed that the distribution of temperature and the intensity of heat flux on the contact surface are continuous, which allow one to determine the mean surface temperature and partition of generated frictional heat [8]. The assumption of these perfect thermal contact conditions is a classical approach, applied in most of the models proposed to date for the frictional heating process in sliding systems [9]. However, these conditions only describe the macroscopic geometry of coupled elements with ideally smooth rubbing surfaces, which does not account well for the tribological phenomena occurring at the sliding interface. This means that the perfect thermal contact conditions at the nominal friction surface do not allow one to model the heating process adequately. Considering the microscopic tribological effects from mechanical and thermal perspectives more closely, contact between two sliding solids is not perfect. Since the friction surfaces are rough rather than smooth, the real contact area is much smaller than the apparent (nominal) contact area [10]. When heat flows across the non-ideal contact zone, temperature discontinuity occurs at the interface. So, precise predictions of temperature fields in a friction zone require the consideration of imperfect thermal contact at the interface [11,12]. Experimental studies show a significant thermal gradient at the macroscopic level between rubbing surfaces of the friction pair elements [13,14]. For instance, in disc braking systems, typically, the values of temperature on the pad’s contact surface are higher than the corresponding values related to the disc [1]. During braking, the pad surface is hotter, since it is in constant friction contact with the disc and it is made of softer material, so it can be more easily plastically deformed and worn than the harder disc [3,15]. Moreover, not only the temperature distribution in a friction couple element but also the heat partition between the sliding components is sensitive to the interfacial boundary conditions [16].

Non-ideal contact is more representative of the heat transfer phenomenon at the interface of coupled bodies sliding against each other. The imperfect thermal contact conditions state that the intensity of heat flux is continuous, according to the law of energy conservation, and there is a gap between temperature values achieved on the contacting surfaces of the sliding elements [9]. This temperature drop is said to be a result of thermal contact resistance existing at the interface [5,17], which is defined as the ratio between the temperature gradient at the contacting surfaces and the heat flux flowing across the interface. Study [16] shows that the value of thermal resistance at the interface of the coupled elements decreases with an increase in relative sliding velocity. The estimation of the thermal contact resistance for a tribosystem made of a semi-infinite foundation (the disc) sliding over the surface of a strip (the braking pad) has been performed in [18]. The inverse of thermal contact resistance is termed heat contact conductance, and it is dependent on numerous factors, such as load, sliding velocity, temperature, and the properties of friction materials [3,19,20,21]. The effect of the thermal contact conductance on the temperature and heat partition at the interface of the disc/pad system during braking has been investigated in [22]. A numerical model has been proposed in [23] to simultaneously determine the heat flux generated by friction, the thermal contact conductance, and the intrinsic heat partition coefficient for the problem of sliding contacts. The experimental validation of the thermal behavior of a system consisting of two hollow cylinders under imperfect thermal sliding contact has been presented in [24]. Godet introduced the third body concept [25,26], i.e., the presence of a third body between friction pair elements, as an explanation for the existence of thermal contact resistance. The formation of a third body in disc/pad contact in a material sense can be defined as an interface zone between contacting surfaces formed from the accumulation of wear debris and contaminants from the surrounding environment, e.g., dust or water [27,28]. A third body has been introduced to an FE model of a contact interface as a thin layer with uniform volumetric heat generation in article [29]. Based on this model, the temperature and the heat distributions in both components of a disc brake system have been evaluated. It was established that the results were primarily sensitive to the third body layer thickness and its conductivity. Another numerical simulation in [15] demonstrated that due to the thermal resistance of a third body, the contact temperatures of the pad were higher and that the increase in the third body’s thickness resulted in a greater temperature gradient between the contacting surfaces of the disc and pad. In another study [1], the temperature fields in the disc and pin have been modeled using a finite element analysis. The authors developed three models using three different approaches, i.e., considering the heat partition ratio, the perfect thermal contact conditions between the sliding surfaces, and the third body concept. The model assuming perfect contact with thermal continuity at the interface was found to better fit the experimental temperature measurements and to be in good agreement with the observed wear occurring at the pin–disc interface. This pin-on-disc tribotest was performed under mild sliding conditions, so the thermal resistance at the interface was low, which was similar to the perfect heat contact. Likewise, by proceeding with the thermal contact conductance to the infinite value in the condition of imperfect thermal contact related to the temperature drop between sliding surfaces, the temperature continuity condition was obtained [9,25,30].

Most analytical studies dealing with thermal analysis during braking processes have been proposed based on the perfect contact assumptions, which is a less realistic but an easier development [9]. However, in some analytical models of transient temperature distributions in the braking systems, the conditions of imperfect thermal contact have been involved. Exact solutions to the non-stationary problems of heat conduction for two homogeneous semi-spaces under non-ideal thermal contact conditions have been obtained in papers [31,32]. A three-dimensional exact solution to determine the mean and flash temperature and the thermal resistance have been proposed in [33]. Problems of non-stationary heat conduction for two sliding semi-spaces under imperfect conditions at the interface have been considered in [34,35] to determine the contact temperatures and heat partition coefficient. The generalized thermal contact conditions have been involved in the problem formulated in [36] to establish the effect of time-dependent thermal contact heat conductance. The analytical solutions to a thermal problem of friction during braking considering heat transfer through the disc/pad interface have been obtained for a strip/semi-space system with the constant intensity of heat generation [37] for a three-element tribosystem (disc/pad/caliper) with time-dependent specific power of friction [38] and for a two-element system during braking with constant deceleration accounting for the convective cooling on a free surface of the pad [39].

The above-mentioned studies concerned tribosystems with friction pair elements made of homogeneous materials. Basically, modern friction systems are more likely to be made of the nonhomogeneous composites, such as functionally graded materials (FGMs) [40]. These materials are characterized by a smooth change in their properties along certain direction(s). The ability to design the continuous spatial distribution of material properties is a great advantage, which makes FGMs widely used in many varied applications [41,42]. The imperfect thermal contact of friction systems with FGM has been studied in some papers. Frictionally excited thermoelastic instability with consideration of the thermal contact resistance at the interface has been considered for a functionally graded layer and a homogeneous half-plane [43], for two sliding FGMs [44], and for a system consisting of an FGM semi-infinite body sliding against a homogeneous half-space [45]. Analytical solutions to the heating problems of thermoelastic semi-space with imperfectly bonded, functionally graded coating have been proposed in [46,47].

A functionally graded strip heated on the friction surface and cooled by convection on the free surface and sliding against the homogeneous semi-space under perfect thermal contact conditions has been considered in our previous study [48]. It was assumed that the strip was made of a two-component FGM with exponentially changing thermal conductivity along its thickness. This paper presents the exact solution to the boundary-value problem of heat conduction for the same friction system with consideration of the thermal contact conductivity at the strip/semi-space interface. Furthermore, asymptotic solutions were also identified for both low and high values of Fourier number. The developed mathematical model allowed for the investigation of the thermal conductance on the contact surface and convective heat exchange with the environment on the temperature distribution.

The results contained in this article are a continuation of our previous research on the use of FGM to reduce the temperature of friction elements [48,49]. In these previous papers, the relevance of this topic was justified, and a broad literature review was presented. In this article, we focused on obtaining an accurate solution to the problem of a friction pair, in which one of the elements is made of FGM (strip) under conditions of imperfect thermal contact of friction. The solutions known to date concern the contact of smooth friction surfaces (perfect thermal contact of friction). Solutions to problems with imperfect thermal contact of friction only concerned homogeneous materials [11,18]. In our work, we fill this research gap.

The proposed research methodology for developing a mathematical model of heat generation due to friction in the strip/semi-space system with imperfect thermal contact of friction consisted of the following stages:

(1) Formulating and obtaining an exact solution to the appropriate boundary-value thermal conductivity problem.
(2) Verification of the received solution.
(3) Obtaining asymptotic solutions for small and large values of the Fourier number.
(4) Numerical analysis for selected materials of the friction pair.
(5) Summary and conclusions.

## 2. Materials and Methods

**Figure 1:**
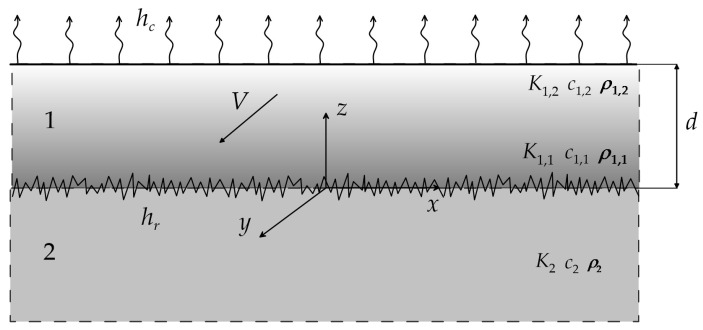
Scheme of the imperfect thermal contact of the strip and the semi-space.

Let us consider the strip/semi-space system heated as a result of the friction on their sliding contact surfaces. The strip 0≤z≤d is made of a two-component functionally graded material (FGM) and the semi-space z≤0 of a homogeneous material (Figure 1).

The coefficient of thermal conductivity of FGM increases exponentially with the distance from the sliding surface of the body:

(1) $$K_{1}(z)=K_{1,1}e^{\gamma^{*}z/d},\;\gamma^{*}=\ln(K_{1,2}K_{1,1}^{-1}),\;0\leq z\leq d,$$

where $K_{1,1}$, $K_{1,2}$, and $K_{2}$—coefficients of thermal conductivity, respectively, of two components of the FGM layer and the half-plane. The thermal friction contact of the strip with the semi-space is imperfect, and the specific friction power remains constant and equal $q_{0}$ during the entire heating process. Also, the coefficients of contact heat conductivity $h_{r}$ through the surfaces z=0 and convective heat exchange $h_{c}$ on the free surface of the strip z=d are constant. At the initial moment t=0, the temperature of the strip and the semi-space is the same and equal $T_{0}$. Other simplifying assumptions can be found in article [50]. It should be emphasized that the transient temperature field T(z,t), −∞<z≤d, t>0 of the system will be found from the following boundary-value problem of heat conduction:

(2) $$\frac{\partial^{2}\Theta^{*}(\zeta ,\tau )}{\partial \zeta^{2}}+\gamma^{*}\frac{\partial \Theta^{*}(\zeta ,\tau )}{\partial \zeta }-e^{-\gamma^{*}\zeta }\frac{\partial \Theta^{*}(\zeta ,\tau )}{\partial \tau }=0,\;0<\zeta <1,\;\tau >0,$$

(3) $$\frac{\partial^{2}\Theta^{*}(\zeta ,\tau )}{\partial \zeta^{2}}-\frac{1}{k^{*}}\frac{\partial \Theta^{*}(\zeta ,\tau )}{\partial \tau }=0,\;\zeta <0,\;\tau >0,$$

(4) $$K^{*}{\frac{\partial \Theta^{*}(\zeta ,\tau )}{\partial \zeta }|}_{\zeta =0^{-}}-{\frac{\partial \Theta^{*}(\zeta ,\tau )}{\partial \zeta }|}_{\zeta =0^{+}}=1,\;\tau >0,$$

(5) $$K^{*}{\frac{\partial \Theta^{*}(\zeta ,\tau )}{\partial \zeta }|}_{\zeta =0^{-}}+{\frac{\partial \Theta^{*}(\zeta ,\tau )}{\partial \zeta }|}_{\zeta =0^{+}}+Bi_{r}[\Theta^{*}(0^{-},\tau )-\Theta^{*}(0^{+},\tau )]=0,\;\tau >0,$$

(6) $${e^{\gamma^{*}}\frac{\partial \Theta^{*}(\zeta ,\tau )}{\partial \zeta }|}_{\zeta =1}+Bi_{c}\;\Theta^{*}(1,\tau )=0,\;\tau >0,$$

(7) $$\Theta^{*}(\zeta ,\tau )\to 0,\;\zeta \to -\infty ,\;\tau >0,$$

(8) $$\Theta^{*}(\zeta ,0)=0,\;\left|\zeta \right|<\infty ,$$

where

(9) $$\zeta =\frac{z}{d},\;\tau =\frac{k_{1}t}{d^{2}},\;K^{*}=\frac{K_{2}}{K_{1,1}},\;k^{*}=\frac{k_{2}}{k_{1}},\;\Theta^{*}=\frac{\Theta }{\Theta_{0}},\;Bi_{r}=\frac{h_{r}d}{K_{1,1}},\;Bi_{c}=\frac{h_{c}d}{K_{1,1}},$$

(10) $$k_{1}=\frac{K_{1,1}}{c_{1}\rho_{1}},\;k_{2}=\frac{K_{2}}{c_{2}\rho_{2}},\;\Theta_{0}=\frac{q_{0}d}{K_{1,1}},$$

$\Theta (z,t)=T(z,t)-T_{0}$—temperature rise; $\rho_{l}$, $c_{l}$—the density and specific heat of materials of the strip (l=1) and the semi-space (l=2).

*Exact solution.* By applying into the initial-boundary problem (2)–(8), the integral Laplace transform [51]:

(11) Θ¯∗(ζ,p)≡L[Θ∗(ζ,τ);p]=∫0∞Θ∗(ζ,τ)e−pτdτ, Rep≥0,

the following boundary problem was received for a system of two ordinary differential equations:

(12) $$\frac{d^{2}{\bar{\Theta }}^{*}(\zeta ,p)}{d\zeta^{2}}+\gamma^{*}\frac{d{\bar{\Theta }}^{*}(\zeta ,\tau )}{d\zeta }-pe^{-\gamma^{*}\zeta }{\bar{\Theta }}^{*}(\zeta ,p)=0,\;0<\zeta <1,$$

(13) $$\frac{d^{2}{\bar{\Theta }}^{*}(\zeta ,p)}{d\zeta^{2}}-\frac{p}{k^{*}}{\bar{\Theta }}^{*}(\zeta ,p)=0,\;\zeta <0,$$

(14) $${K^{*}\frac{d{\bar{\Theta }}^{*}(\zeta ,p)}{d\zeta }|}_{\zeta =0^{-}}-{\frac{d{\bar{\Theta }}^{*}(\zeta ,p)}{d\zeta }|}_{\zeta =0^{+}}=\frac{1}{p},$$

(15) $${K^{*}\frac{d{\bar{\Theta }}^{*}(\zeta ,p)}{d\zeta }|}_{\zeta =0^{-}}+{\frac{d{\bar{\Theta }}^{*}(\zeta ,p)}{d\zeta }|}_{\zeta =0^{+}}+Bi_{r}[{\bar{\Theta }}^{*}(0^{-},\tau )-{\bar{\Theta }}^{*}(0^{+},\tau )]=0,$$

(16) $${e^{\gamma^{*}}\frac{d{\bar{\Theta }}^{*}(\zeta ,p)}{d\zeta }|}_{\zeta =1}+Bi_{c}\;{\bar{\Theta }}^{*}(1,p)=0,$$

(17) $${\bar{\Theta }}^{*}(\zeta ,p)\to 0,\;\zeta \to -\infty .$$

Using the methodology described in article [50], the solutions to the problem (12)–(17) have the following form:

(18) $${\bar{\Theta }}^{*}(\zeta ,p)=\frac{e^{-\frac{1}{2}\gamma^{*}\zeta }}{p\sqrt{p}}\left(\varepsilon +\frac{Bi_{r}}{\sqrt{p}}\right)\frac{\Delta_{1}(\zeta ,p)}{\;\Delta (p)},\;0\leq \zeta \leq 1,$$

(19) $${\bar{\Theta }}^{*}(\zeta ,p)=\frac{\Delta_{2}(\zeta ,p)}{p\sqrt{p}\;\Delta (p)},\;\zeta \leq 0,$$

where

(20) $$\begin{array}{l} \Delta (p)=A_{1}(p)\left[\left(2\varepsilon +\frac{Bi_{r}}{\sqrt{p}}\right)I_{0}\left(\frac{2}{\gamma^{*}}\sqrt{p}\right)+\varepsilon \frac{Bi_{r}}{\sqrt{p}}\;I_{1}\left(\frac{2}{\gamma^{*}}\sqrt{p}\right)\right]-\\ \;\;\;\;\;\;\;\;\;\;\;\;\;-B_{1}(p)\left[\left(2\varepsilon +\frac{Bi_{r}}{\sqrt{p}}\right)K_{0}\left(\frac{2}{\gamma^{*}}\sqrt{p}\right)-\varepsilon \frac{Bi_{r}}{\sqrt{p}}\;K_{1}\left(\frac{2}{\gamma^{*}}\sqrt{p}\right)\right]\;, \end{array}$$

(21) $$\Delta_{1}(\zeta ,p)=A_{1}(p{)I}_{1}\left(\frac{2}{\gamma^{*}}e^{-\frac{1}{2}\gamma^{*}\zeta }\sqrt{p}\right)+B_{1}(p{)K}_{1}\left(\frac{2}{\gamma^{*}}e^{-\frac{1}{2}\gamma^{*}\zeta }\sqrt{p}\right),\;\Delta_{2}(\zeta ,p)=A_{2}(p)e^{\sqrt{\frac{p}{k^{*}}}\;\zeta },$$

(22) $$A_{1}(p)=K_{0}\left(\frac{2}{\gamma^{*}}e^{-\frac{1}{2}\gamma^{*}}\sqrt{p}\right)+Bi_{c}\frac{e^{-\frac{1}{2}\gamma^{*}}}{\sqrt{p}}K_{1}\left(\frac{2}{\gamma^{*}}e^{-\frac{1}{2}\gamma^{*}}\sqrt{p}\right),$$

(23) $$B_{1}(p)=I_{0}\left(\frac{2}{\gamma^{*}}e^{-\frac{1}{2}\gamma^{*}}\sqrt{p}\right)-Bi_{c}\frac{e^{-\frac{1}{2}\gamma^{*}}}{\sqrt{p}}I_{1}\left(\frac{2}{\gamma^{*}}e^{-\frac{1}{2}\gamma^{*}}\sqrt{p}\right),$$

(24) $$\;\;\;\;\;\;\;\;\;A_{2}(p)=A_{1}(p)\left[I_{0}\left(\frac{2}{\gamma^{*}}\sqrt{p}\right)+\frac{Bi_{r}}{\sqrt{p}}\;I_{1}\left(\frac{2}{\gamma^{*}}\sqrt{p}\right)\right]-\;-B_{1}(p)\left[K_{0}\left(\frac{2}{\gamma^{*}}\sqrt{p}\right)-\varepsilon \frac{Bi_{r}}{\sqrt{p}}\;K_{1}\left(\frac{2}{\gamma^{*}}\sqrt{p}\right)\right],$$

(25) $$\varepsilon =\frac{K^{*}}{\sqrt{k^{*}}},$$

$I_{n}(x)$, $K_{n}(x)$—modified Bessel functions of the *n*th order n=0,1, respectively, of the first and the second kind [52].

Applying to the transformed solution (18)–(25) the inverse Laplace transform [51]:

(26) Θ∗(ζ,τ)≡L−1[Θ¯∗(ζ,p); τ]=12πi∫ω−i ∞ω+i ∞Θ¯∗(ζ,p)epτdp, ω≡Rep>0, i≡−1,

and performing the integration on the plane of the complex variable p in line with the methodology presented in the study [49] using the relations [52]:

(27) $$I_{0}(\pm ix)=J_{0}(x),\;K_{0}(\pm ix)=-0.5\pi [Y_{0}(x)\pm iJ_{0}\left(x\right)],$$

(28) $$I_{1}(\pm ix)=\pm iJ_{1}(ix),\;K_{1}(\pm ix)=0.5\pi [J_{1}(x)\pm iY_{1}\left(x\right)],$$

($J_{n}(x)$ and $Y_{n}(x)$—Bessel functions of the *n*th order n=0,1, respectively, of the first and the second kind), dimensionless temperature rises in the strip and semi-space were found in the following form:

(29) $$\Theta^{*}(\zeta ,\tau )=\vartheta_{1}(\zeta )-\frac{2}{\pi }\varepsilon \;e^{-\frac{1}{2}\gamma^{*}\zeta }\int_{0}^{\infty }F(x)G_{1}(\zeta ,x)e^{-x^{2}\tau }dx,\;0\leq \zeta \leq 1,\;\tau \geq 0,$$

(30) $$\Theta^{*}(\zeta ,\tau )=\vartheta_{2}-\frac{2}{\pi }\int_{0}^{\infty }F(x)G_{2}(\zeta ,x)e^{-x^{2}\tau }dx,\;\zeta \leq 0,\;\tau \geq 0,$$

where

(31) $$\vartheta_{1}(\zeta )=\frac{1}{Bi_{c}}+\frac{1}{\gamma^{*}}(e^{-\gamma^{*}\zeta }-e^{-\gamma^{*}}),\;\vartheta_{2}=\frac{1}{Bi_{r}}+\frac{1}{Bi_{c}}+\frac{1}{\gamma^{*}}(1-e^{-\gamma^{*}}),$$

(32) $$F(x)=\frac{\left[\Psi (x)-x\;Bi_{r}^{-1}\Phi (x)\right]}{x^{2}\{{\left[\Phi (x)\right]}^{2}+{\left[\varepsilon \;\Psi (x)\right]}^{2}\}},$$

(33) $$G_{1}(\zeta ,x)=J(x)Y_{1}\left(\frac{2}{\gamma^{*}}e^{-\frac{1}{2}\gamma^{*}\zeta }x\right)-Y(x)J_{1}\left(\frac{2}{\gamma^{*}}e^{-\frac{1}{2}\gamma^{*}\zeta }x\right),$$

(34) $$G_{2}(\zeta ,x)=\varepsilon \;\Psi (x)\cos\left(\frac{\zeta }{\sqrt{k^{*}}}x\right)-\Phi (x)\sin\left(\frac{\zeta }{\sqrt{k^{*}}}x\right),$$

(35) $$\Phi (x)=J(x)Y_{0}\left(\frac{2}{\gamma^{*}}x\right)-Y(x)J_{0}\left(\frac{2}{\gamma^{*}}x\right),$$

(36) $$\Psi (x)=J(x)\left[Y_{1}\left(\frac{2}{\gamma^{*}}x\right)+\frac{2x}{Bi_{r}}Y_{0}\left(\frac{2}{\gamma^{*}}x\right)\right]-Y(x)\left[J_{1}\left(\frac{2}{\gamma^{*}}x\right)+\frac{2x}{Bi_{r}}J_{0}\left(\frac{2}{\gamma^{*}}x\right)\right],$$

(37) $$J(x)=J_{0}\left(\frac{2}{\gamma^{*}}e^{-\frac{1}{2}\gamma^{*}}x\right)-Bi_{c}\frac{e^{-\frac{1}{2}\gamma^{*}}}{x}J_{1}\left(\frac{2}{\gamma^{*}}e^{-\frac{1}{2}\gamma^{*}}x\right),$$

(38) $$Y(x)=Y_{0}\left(\frac{2}{\gamma^{*}}e^{-\frac{1}{2}\gamma^{*}}x\right)-Bi_{c}\frac{e^{-\frac{1}{2}\gamma^{*}}}{x}Y_{1}\left(\frac{2}{\gamma^{*}}e^{-\frac{1}{2}\gamma^{*}}x\right).$$

Substituting ζ=0 in the solutions (29)–(38), the following expressions were used to estimate temperature on the sliding surfaces of the strip ($z=0^{+}$) and of the semi-space ($z=0^{-}$) for imperfect thermal contact of friction:

(39) $$\Theta^{*}(0^{+},\tau )=\vartheta_{0}-\frac{2}{\pi }\varepsilon \int_{0}^{\infty }F(x)\Psi_{0}(x)e^{-x^{2}\tau }dx,\;\tau \geq 0,$$

(40) $$\Theta^{*}(0^{-},\tau )=(Bi_{r}^{-1}+\vartheta_{0})-\frac{2}{\pi }\varepsilon \int_{0}^{\infty }F(x)\Psi (x)e^{-x^{2}\tau }dx,\;\tau \geq 0,$$

where

(41) $$\vartheta_{0}\equiv \vartheta_{1}(0)=\frac{1}{Bi_{c}}+\frac{1}{\gamma^{*}}(1-e^{-\gamma^{*}}),$$

(42) $$\Psi_{0}(x)\equiv G_{1}(0,x)=J(x)Y_{1}\left(\frac{2}{\gamma^{*}}x\right)-Y(x)J_{1}\left(\frac{2}{\gamma^{*}}x\right).$$

In the case of ideal heat contact of the strip and the semi-space ($Bi_{r}\to \infty$), Equations (36) and (42) give $\Psi (x)=\Psi_{0}(x)$, and from solutions (39) and (40), it follows that the temperature of the friction surfaces is the same and equal [50]:

(43) $$\Theta^{*}(0^{+},\tau )=\Theta^{*}(0^{-},\tau )\equiv \Theta^{*}(\tau )=\vartheta_{0}-\frac{2}{\pi }\varepsilon \int_{0}^{\infty }F_{0}(x)\Psi_{0}(x)e^{-x^{2}\tau }dx,\;\tau \geq 0,$$

where

(44) $$F_{0}(x)=\frac{\Psi_{0}(x)}{x^{2}\{{\left[\Phi (x)\right]}^{2}+{\left[\varepsilon \;\Psi_{0}(x)\right]}^{2}\}}.$$

Solutions (29)–(38) have been obtained for constant-value $q_{0}$ of the specific friction power. The form of these expressions allows for the usage of Duhamel’s theorem in order to obtain corresponding solutions to this problem for time-dependent specific friction power. In this way, for the case of the specific friction power linearly decreasing from the nominal value ${\hat{q}}_{0}=2q_{0}$ at the initial moment t=0 to zero at the final moment $t=t_{s}$, the dimensionless temperature rise ${\hat{\Theta }}^{*}(\zeta ,\tau )$, based on Duhamel’s formula, was sought as [53]:

(45) $${\hat{\Theta }}^{*}(\zeta ,\tau )=\frac{\partial }{\partial \tau }\int_{0}^{\tau }q^{*}(\tau -s)\Theta^{*}(\zeta ,s)ds,\;\zeta \geq 0,\;0\leq \tau \leq \tau_{s},$$

where

(46) $$q^{*}(\tau )=1-\tau \;\tau_{s}^{-1},0\leq \tau \leq \tau_{s},\;\tau_{s}=k_{1}t_{s}d^{-2},$$

and $\Theta^{*}(\zeta ,\tau )$ is the dimensionless temperature rise (29)–(38) achieved for $q^{*}(\tau )=q_{0},\;\tau \geq 0$.

After substituting under the integral sign in the right side of Formula (45) functions $\Theta^{*}(\zeta ,\tau )$ (29), (30) and $q^{*}(\tau )$ (53), the following was found:

(47) $${\hat{\Theta }}^{*}(\zeta ,\tau )=\vartheta_{1}(\zeta )q^{*}(\tau )-\frac{2}{\pi }\varepsilon \;e^{-\frac{1}{2}\gamma^{*}\zeta }\int_{0}^{\infty }F(x)G_{1}(\zeta ,x)P(\tau ,x)dx,\;\;0\leq \zeta \leq 1,\;0\leq \tau \leq \tau_{s},$$

(48) $${\hat{\Theta }}^{*}(\zeta ,\tau )=\vartheta_{2}q^{*}(\tau )-\frac{2}{\pi }\int_{0}^{\infty }F(x)G_{2}(\zeta ,x)P(\tau ,x)dx,\;\zeta \leq 0,\;0\leq \tau \leq \tau_{s}’$$

where

(49) $$P(\tau ,x)=e^{-x^{2}\tau }-\frac{1}{x^{2}\tau_{s}}(1-e^{-x^{2}\tau }),$$

and the rest of the functions have been determined in Equations (31)–(28).

*Verification of the solution.* Validation of the obtained exact solution (29)–(38) was carried out by checking the fulfillment of boundary conditions (4)–(6). For this purpose, on account of the derivatives [52]:

(50) $$J_{1}^{\prime }(x)=J_{0}(x)-x^{-1}J_{1}(x),\;Y_{1}^{\prime }(x)=Y_{0}(x)-x^{-1}Y_{1}(x),$$

and from solutions (29) and (30), the dimensionless intensities of heat fluxes in the strip and in the semi-space were found:

(51) $$\frac{\partial \Theta^{*}(\zeta ,\tau )}{\partial \zeta }=-e^{-\gamma^{*}\zeta }+\frac{2}{\pi }\varepsilon \;e^{-\gamma^{*}\zeta }\int_{0}^{\infty }xF(x){\hat{G}}_{1}(\zeta ,x)e^{-x^{2}\tau }dx,\;0\leq \zeta \leq 1,\;\tau \geq 0,$$

(52) $$K^{*}\frac{\partial \Theta^{*}(\zeta ,\tau )}{\partial \zeta }=\frac{2}{\pi }\varepsilon \int_{0}^{\infty }xF(x){\hat{G}}_{2}(\zeta ,x)e^{-x^{2}\tau }dx,\;\zeta \leq 0,\;\tau \geq 0,$$

where

(53) $${\hat{G}}_{1}(\zeta ,x)=J(x)Y_{0}\left(\frac{2}{\gamma^{*}}e^{-\frac{1}{2}\gamma^{*}\zeta }x\right)-Y(x)J_{0}\left(\frac{2}{\gamma^{*}}e^{-\frac{1}{2}\gamma^{*}\zeta }x\right),$$

(54) $${\hat{G}}_{2}(\zeta ,x)=\varepsilon \;\Psi (x)\sin\left(\frac{\zeta }{\sqrt{k^{*}}}x\right)+\Phi (x)\cos\left(\frac{\zeta }{\sqrt{k^{*}}}x\right).$$

On the sliding surfaces $\zeta =0^{\pm }$, Equations (51)–(54) yield:

(55) $${q_{1}^{*}(\tau )\equiv -\frac{\partial \Theta^{*}(\zeta ,\tau )}{\partial \zeta }|}_{\zeta =0^{+}}=1-\frac{2}{\pi }\varepsilon \int_{0}^{\infty }xF(x)\Phi (x)e^{-x^{2}\tau }dx,\;\tau \geq 0,$$

(56) $$q_{2}^{*}(\tau )\equiv {K^{*}\frac{\partial \Theta^{*}(\zeta ,\tau )}{\partial \zeta }|}_{\zeta =0^{-}}=\frac{2}{\pi }\varepsilon \int_{0}^{\infty }xF(x)\Phi (x)e^{-x^{2}\tau }dx,\;\tau \geq 0,$$

where functions F(x) and Φ(x) have the forms, respectively, of (32) and (35). The addition of Formulas (55) and (56) confirms the fulfillment of boundary condition (4). However, after subtracting them, it was found that

(57) $${K^{*}\frac{\partial \Theta^{*}(\zeta ,\tau )}{\partial \zeta }|}_{\zeta =0^{-}}+{\frac{\partial \Theta^{*}(\zeta ,\tau )}{\partial \zeta }|}_{\zeta =0^{+}}=-1+\frac{4}{\pi }\varepsilon \int_{0}^{\infty }xF(x)\Phi (x)e^{-x^{2}\tau }dx,\;\tau \geq 0.$$

On the other hand, subtracting Equations (39) and (40) results in

(58) $$\Theta^{*}(0^{-},\tau )-\Theta^{*}(0^{+},\tau )=\frac{1}{Bi_{r}}-\frac{2}{\pi }\int_{0}^{\infty }F(x)[\Psi (x)-\varepsilon \;\Psi_{0}(x)]e^{-x^{2}\tau }dx,\;\tau \geq 0.$$

Considering the form of the functions Φ(x) (35), Ψ(x) (36), and $\Psi_{0}(x)$ (42), it was established that

(59) $$\Psi (x)-\varepsilon \;\Psi_{0}(x)=2\varepsilon \;Bi_{r}^{-1}\;x\Phi (x).$$

Then, based on the expression (58) and (59), the following was received:

(60) $$Bi_{r}[\Theta^{*}(0^{+},\tau )-\Theta^{*}(0^{-},\tau )]=1-\frac{4}{\pi }\varepsilon \int_{0}^{\infty }xF(x)\Phi (x)e^{-x^{2}\tau }dx,\;\tau \geq 0.$$

The result of the addition of Formulas (57) and (60) confirms that boundary condition (5) is met.

The dimensionless intensity of heat flux on the free surface of the strip ζ=1, from Equations (52) and (54), was determined:

(61) $${\frac{\partial \Theta^{*}(\zeta ,\tau )}{\partial \zeta }|}_{\zeta =1}=-e^{-\gamma^{*}}-\varepsilon \;e^{-\gamma^{*}}\frac{2}{\pi }\int_{0}^{\infty }xF(x){\hat{G}}_{1}(1,x)e^{-x^{2}\tau }dx,\;\tau \geq 0,$$

where

(62) $${\hat{G}}_{1}(1,x)=J(x)Y_{0}\left(\frac{2}{\gamma^{*}}e^{-\frac{1}{2}\gamma^{*}}x\right)-Y(x)J_{0}\left(\frac{2}{\gamma^{*}}e^{-\frac{1}{2}\gamma^{*}}x\right).$$

Then, from solution (29), a suitable dimensionless temperature rise was found:

(63) $$\Theta^{*}(1,\tau )=\frac{1}{Bi_{c}}-\varepsilon \;e^{-\frac{1}{2}\gamma^{*}}\frac{2}{\pi }\int_{0}^{\infty }F(x)G_{1}(1,x)e^{-x^{2}\tau }dx,\;\tau \geq 0,$$

where, based on Equation (33), the following was obtained:

(64) $$G_{1}(1,x)=J(x)Y_{1}\left(\frac{2}{\gamma^{*}}e^{-\frac{1}{2}\gamma^{*}}x\right)-Y(x)J_{1}\left(\frac{2}{\gamma^{*}}e^{-\frac{1}{2}\gamma^{*}}x\right).$$

From relations (61) and (63), it follows that

(65) $${e^{\gamma^{*}}\frac{\partial \Theta^{*}(\zeta ,\tau )}{\partial \zeta }|}_{\zeta =1}+Bi_{c}\Theta^{*}(1,\tau )=\frac{2}{\pi }\varepsilon \int_{0}^{\infty }F(x)[x{\hat{G}}_{1}(1,x)-Bi_{c}e^{-\frac{1}{2}\gamma^{*}}G_{1}(1,x)]e^{-x^{2}\tau }dx,\;\;\tau \geq 0.$$

Taking into account forms of functions ${\hat{G}}_{1}(1,x)$ (62) and $G_{1}(1,x)$ (64), the following was achieved:

(66) $$\begin{array}{l} x{\hat{G}}_{1}(1,x)-Bi_{c}e^{-\frac{1}{2}\gamma^{*}}G_{1}(1,x)=J(x)\left[xY_{0}\left(\frac{2}{\gamma^{*}}e^{-\frac{1}{2}\gamma^{*}}x\right)-Bi_{c}e^{-\frac{1}{2}\gamma^{*}}Y_{1}\left(\frac{2}{\gamma^{*}}e^{-\frac{1}{2}\gamma^{*}}x\right)\right]-\\ \;\;\;\;\;\;\;\;\;\;\;\;\;\;\;\;\;\;\;\;\;\;\;\;\;\;\;\;\;\;\;\;\;\;\;\;\;\;\;\;\;\;\;\;\;\;\;\;\;\;-Y(x)\left[xJ_{0}\left(\frac{2}{\gamma^{*}}e^{-\frac{1}{2}\gamma^{*}}x\right)-Bi_{c}e^{-\frac{1}{2}\gamma^{*}}J_{1}\left(\frac{2}{\gamma^{*}}e^{-\frac{1}{2}\gamma^{*}}x\right)\right]=\\ \;\;\;\;\;\;\;\;\;\;\;\;\;\;\;\;\;\;\;\;\;\;\;\;\;\;\;\;\;\;\;\;\;\;\;\;\;\;\;\;\;\;\;\;\;\;\;\;\;\;=x[J(x)Y(x)-Y(x)J(x)]=0, \end{array}$$

which confirms the fulfillment of boundary condition (6).

The fulfillment of the boundary condition (7) and the initial condition (8) were taken into account when obtaining the solution (18)–(25) in the space of the Laplace integral transform.

*Asymptotic solutions*. Small values of the Fourier number τ (large values of the Laplace transform parameter p). Bearing in mind Formulas (18)–(24) and the asymptotes of the modified Bessel functions for large values of the argument [52]:

(67) $$I_{n}(x)≅\frac{e^{x}}{\sqrt{2\pi x}},\;K_{n}(x)≅\sqrt{\frac{\pi }{2x}}\;e^{-x},\;n=0,\;1,$$

it was established that the solution in the Laplace transform space has the following form:

(68) $${\bar{\Theta }}^{*}(\zeta ,p)≅\frac{1}{2}e^{-\frac{1}{4}\gamma^{*}\zeta }\left[\frac{e^{-\zeta_{1}\sqrt{p}}}{p(\alpha +\sqrt{p})}+\frac{Bi_{r}}{\varepsilon }\frac{e^{-\zeta_{1}\sqrt{p}}}{p\sqrt{p}(\alpha +\sqrt{p})}\right],\;\;0\leq \zeta <1,$$

(69) $${\bar{\Theta }}^{*}(\zeta ,p)≅\frac{1}{2\varepsilon }\left[\frac{e^{-\zeta_{2}\sqrt{p}}}{p(\alpha +\sqrt{p})}+Bi_{r}\frac{e^{-\zeta_{2}\sqrt{p}}}{p\sqrt{p}(\alpha +\sqrt{p})}\right],\;\;\zeta \leq 0,$$

where

(70) $$\alpha =\frac{\left(1+\varepsilon \right)}{2\varepsilon }Bi_{r},\;\zeta_{1}=\frac{2}{\gamma^{*}}(1-e^{-\frac{1}{2}\gamma^{*}\zeta }),\zeta_{2}=\frac{\left|\zeta \right|}{\sqrt{k^{*}}}.$$

Using relations [54]:

(71) $$L^{-1}\left[\frac{\alpha e^{-\zeta_{l}\sqrt{p}}}{p(\alpha +\sqrt{p})};\;\tau \right]=\mathrm{erfc}\left(\frac{\zeta_{l}}{2\sqrt{\tau }}\right)-e^{\alpha^{2}\tau +\alpha \zeta_{l}}\mathrm{erfc}\left(\frac{\zeta_{l}}{2\sqrt{\tau }}+\alpha \sqrt{\tau }\right),\;l=1,2,$$

(72) $$\begin{array}{l} L^{-1}\left[\frac{\alpha e^{-\zeta_{l}\sqrt{p}}}{p\sqrt{p}(\alpha +\sqrt{p})};\;\tau \right]=2\sqrt{\frac{\tau }{\pi }}\;e^{-{\left(\frac{\zeta_{l}}{2\sqrt{\tau }}\right)}^{2}}-\left(\zeta_{l}+\frac{1}{\alpha }\right)\mathrm{erfc}\left(\frac{\zeta_{l}}{2\sqrt{\tau }}\right)+\\ \;\;\;\;\;\;\;\;\;\;\;\;\;\;\;\;\;\;\;\;\;\;\;\;\;\;\;\;\;\;\;\;\;\;\;\;\;\;\;\;\;\;\;\;\;\;\;\;\;\;\;\;\;\;\;\;\;\;\;+\frac{1}{\alpha }e^{\alpha^{2}\tau +\alpha \zeta_{l}}\mathrm{erfc}\left(\frac{\zeta_{l}}{2\sqrt{\tau }}+\alpha \sqrt{\tau }\right),\;l=1,2, \end{array}$$

where erfc(x)=1−erf(x), erf(x)—Gauss error function [52], from Equations (68)–(70), yields:

(73) $$\Theta^{*}(\zeta ,\tau )=\frac{e^{-\frac{1}{4}\gamma^{*}\zeta }}{\left(1+\varepsilon \right)}[2\sqrt{\frac{\tau }{\pi }}\;e^{-\left(\frac{\zeta_{1}}{2\sqrt{\tau }}\right)}{}^{2}-\left(\zeta_{1}+\frac{1}{\alpha }-\frac{\varepsilon }{Bi_{r}}\right)\mathrm{erfc}\left(\frac{\zeta_{1}}{2\sqrt{\tau }}\right)+\;\;\;\;\;\;\;\;\;\;\;\;\;\;+\left(\frac{1}{\alpha }-\frac{\varepsilon }{Bi_{r}}\right)e^{\alpha^{2}\tau +\alpha \zeta_{1}}\mathrm{erfc}\left(\frac{\zeta_{1}}{2\sqrt{\tau }}+\alpha \sqrt{\tau }\right)],\;0\leq \zeta \leq 1,\;0\leq \tau <<1,$$

(74) $$\Theta^{*}(\zeta ,\tau )=\frac{1}{\left(1+\varepsilon \right)}[2\sqrt{\frac{\tau }{\pi }}\;e^{-\left(\frac{\zeta_{2}}{2\sqrt{\tau }}\right)}{}^{2}-\left(\zeta_{2}+\frac{1}{\alpha }-\frac{1}{Bi_{r}}\right)\mathrm{erfc}\left(\frac{\zeta_{2}}{2\sqrt{\tau }}\right)+\;\;\;\;\;\;\;\;\;\;\;\;\;\;+\left(\frac{1}{\alpha }-\frac{1}{Bi_{r}}\right)e^{\alpha^{2}\tau +\alpha \zeta_{2}}\mathrm{erfc}\left(\frac{\zeta_{2}}{2\sqrt{\tau }}+\alpha \sqrt{\tau }\right)],\;\zeta \leq 0,\;0\leq \tau <<1.$$

Proceeding with Equations (73) and (74) to the limit $Bi_{r}\to \infty$, the known asymptotic solution was obtained, related to the perfect thermal contact between the FGM strip and homogeneous semi-space [53]:

(75) $$\Theta^{*}(\zeta ,\tau )≅\frac{2\sqrt{\tau }}{\left(1+\varepsilon \right)}e^{-\frac{1}{4}\gamma^{*}\zeta }\mathrm{ierfc}\left(\frac{\zeta_{1}}{2\sqrt{\tau }}\right),\;0\leq \zeta \leq 1,\;0\leq \tau <<1,$$

(76) $$\Theta^{*}(\zeta ,\tau )≅\frac{2\sqrt{\tau }}{(1+\varepsilon )}\mathrm{ierfc}\left(\frac{\zeta_{2}}{2\sqrt{\tau }}\right),\;\zeta \leq 0,\;0\leq \tau <<1,$$

where $\mathrm{ierfc}(x)=\pi^{-0.5}e^{-x^{2}}-x\;\mathrm{erfc}(x)$. From Equations (75) and (76), it follows that the temperature of the friction surfaces $\zeta =0^{\pm }$ ($\zeta_{1}=\zeta_{2}=0$) in this case is the same:

(77) $$\Theta^{*}(0^{+},\tau )=\Theta^{*}(0^{-},\tau )=\Theta^{*}(\tau )≅\frac{2}{\left(1+\varepsilon \right)}\sqrt{\frac{\tau }{\pi }}.$$

For the homogeneous material of the strip ($\gamma^{*}\to 0$) from the second formula from Equation (70), it follows that $\zeta_{1}\to \zeta$ and solution (80) has the known form [37]:

(78) $$\Theta^{*}(\zeta ,\tau )=\frac{1}{\left(1+\varepsilon \right)}[2\sqrt{\frac{\tau }{\pi }}\;e^{-\left(\frac{\zeta }{2\sqrt{\tau }}\right)}{}^{2}-\left(\zeta +\frac{1}{\alpha }-\frac{\varepsilon }{Bi_{r}}\right)\mathrm{erfc}\left(\frac{\zeta }{2\sqrt{\tau }}\right)+\;\;\;\;\;\;\;\;\;\;\;\;\;\;+\left(\frac{1}{\alpha }-\frac{\varepsilon }{Bi_{r}}\right)e^{\alpha^{2}\tau +\alpha \zeta }\mathrm{erfc}\left(\frac{\zeta }{2\sqrt{\tau }}+\alpha \sqrt{\tau }\right)],\;0\leq \zeta \leq 1,\;0\leq \tau <<1,$$

and solution (74) for semi-space remains without changes, as it is independent on parameter $\gamma^{*}$.

It should be noted that the form of asymptotic solutions (73) and (74) shows that at the beginning of the friction heating process, the effect of convection cooling of the exposed surface of the strip on the temperature of both bodies is negligible, and the gradient nature of the material only affects the temperature of the strip.

High values of Fourier number τ (small values of the Laplace parameter p). Considering the behavior of modified Bessel functions at small argument values [52]:

(79) $$I_{0}(x)≅1,\;I_{1}(x)≅0.5x,\;K_{0}(x)≅-\ln(x),\;K_{1}(x)≅x^{-1},$$

in Equations (18)–(25), the following was found:

(80) $${\bar{\Theta }}^{*}(\zeta ,p)≅\phi^{*}(\zeta )\left[\frac{1}{p(a+\sqrt{p})}+\frac{\varepsilon }{Bi_{r}}\frac{1}{\sqrt{p}(a+\sqrt{p})}\right],\;\;0\leq \zeta \leq 1,$$

(81) $${\bar{\Theta }}^{*}(\zeta ,p)≅\left(\frac{1}{\varepsilon }-\frac{a}{Bi_{r}}\right)\frac{e^{-\zeta_{2}\sqrt{p}}}{p(a+\sqrt{p})},\;\;\zeta \leq 0,$$

where

(82) $$a=\frac{\gamma^{*}Bi_{r}Bi_{c}}{\varepsilon (\phi_{0}Bi_{r}+2\gamma^{*}Bi_{c})},\;\phi^{*}(\zeta )=\frac{\phi (\zeta )Bi_{r}}{\varepsilon (\phi_{0}Bi_{r}+2\gamma^{*}Bi_{c})},$$

(83) $$\phi (\zeta )=\gamma^{*}+Bi_{c}(e^{-\gamma^{*}\zeta }-e^{-\gamma^{*}}),\;\phi_{0}\equiv \phi (0)=\gamma^{*}+Bi_{c}(1-e^{-\gamma^{*}}),$$

and parameter $\zeta_{2}$ is defined in the last formula of Equation (70). Taking into account relation (71) and the following [54]:

(84) $$L^{-1}\left[\frac{1}{\sqrt{p}(a+\sqrt{p})};\tau \right]=e^{a^{2}\tau }\mathrm{erfc}(a\sqrt{\tau }),\;L^{-1}\left[\frac{a}{p(a+\sqrt{p})};\tau \right]=1-e^{a^{2}\tau }\mathrm{erfc}(a\sqrt{\tau }),$$

asymptotes of dimensionless temperature rise for high values of Fourier number τ were found in the following form:

(85) $$\Theta^{*}(\zeta ,\tau )≅\phi^{*}(\zeta )\left[\frac{1}{a}-\left(\frac{1}{a}-\frac{\varepsilon }{Bi_{r}}\right)\right]e^{a^{2}\tau }\mathrm{erfc}(a\sqrt{\tau }),\;0\leq \zeta \leq 1,\;\tau >>1,$$

(86) $$\Theta^{*}(\zeta ,\tau )≅\left(\frac{1}{a\varepsilon }-\frac{1}{Bi_{r}}\right)\left[\mathrm{erfc}\left(\frac{\zeta_{2}}{2\sqrt{\tau }}\right)-e^{a^{2}\tau +a\zeta_{2}}\mathrm{erfc}\left(\frac{\zeta_{2}}{2\sqrt{\tau }}+a\sqrt{\tau }\right)\right],\;\;\zeta \leq 0,\;\tau >>1.$$

In the case of perfect thermal contact of friction ($Bi_{r}\to \infty$) from Formula (82), it follows that

(87) $$a=\frac{\gamma^{*}Bi_{c}}{\varepsilon \phi_{0}},\;\phi^{*}(\zeta )=\frac{\phi (\zeta )}{\varepsilon \phi_{0}},$$

and solutions (85), (86) will take the form [50]

(88) $$\Theta^{*}(\zeta ,\tau )≅\frac{\phi (\zeta )}{\gamma^{*}Bi_{c}}[1-e^{a^{2}\tau }\mathrm{erfc}(a\sqrt{\tau })],\;0\leq \zeta \leq 1,\;\tau >>1,$$

(89) $$\Theta^{*}(\zeta ,\tau )≅\frac{1}{a\varepsilon }\left[\mathrm{erfc}\left(\frac{\zeta_{2}}{2\sqrt{\tau }}\right)-e^{a^{2}\tau +a\zeta_{2}}\mathrm{erfc}\left(\frac{\zeta_{2}}{2\sqrt{\tau }}+a\sqrt{\tau }\right)\right],\;\;\zeta \leq 0,\;\tau >>1.$$

Considering the limit

(90) $$\underset{\gamma^{*}\to 0}{\lim}\frac{\gamma^{*}}{\phi_{0}}=\frac{Bi_{c}}{\varepsilon (1+Bi_{c})},\;\underset{\gamma^{*}\to 0}{\lim}\frac{\phi (\zeta )}{\gamma^{*}}=1+Bi_{c}(1-\zeta ),$$

from Equation (88), the dimensionless temperature rises for ideal heat contact between the layer and the half-plane were obtained:

(91) $$\Theta^{*}(\zeta ,\tau )≅\left[\frac{1+Bi_{c}(1-\zeta )}{Bi_{c}}\right][1-e^{a^{2}\tau }\mathrm{erfc}(a\sqrt{\tau })],\;0\leq \zeta \leq 1,\;\tau >>1,$$

(92) $$\Theta^{*}(\zeta ,\tau )≅\frac{\left(1+Bi_{c}\right)}{Bi_{c}}\left[\mathrm{erfc}\left(\frac{\zeta_{2}}{2\sqrt{\tau }}\right)-e^{a^{2}\tau +a\zeta_{2}}\mathrm{erfc}\left(\frac{\zeta_{2}}{2\sqrt{\tau }}+a\sqrt{\tau }\right)\right],\;\;\zeta \leq 0,\;\tau >>1,$$

where

(93) $$a=\frac{Bi_{c}}{\varepsilon (1+Bi_{c})}.$$

On the other hand, proceeding with Formulas (82) and (83) to the limit $\gamma^{*}\to 0$, the following was found:

(94) $$a=\frac{Bi_{r}Bi_{c}}{\varepsilon [(1+Bi_{c})Bi_{r}+2Bi_{c}]},\;\phi^{*}(\zeta )=\frac{[1+(1-\zeta )Bi_{c}]Bi_{r}}{\varepsilon [(1+Bi_{c})Bi_{r}+2Bi_{c}]},$$

and from solutions (85) and (86), the following was obtained:

(95) $$\Theta^{*}(\zeta ,\tau )≅\left[\frac{1+Bi_{c}(1-\zeta )}{Bi_{c}}\right]\left\{1-\left[\frac{(1+Bi_{c})Bi_{r}+Bi_{c}}{(1+Bi_{c})Bi_{r}+2Bi_{c}}\right]e^{a^{2}\tau }\mathrm{erfc}(a\sqrt{\tau })\right\},\;0\leq \zeta \leq 1,\;\tau >>1,$$

(96) $$\Theta^{*}(\zeta ,\tau )≅\frac{\left[(1+Bi_{c})Bi_{r}+Bi_{c}\right]}{Bi_{r}Bi_{c}}\left[\mathrm{erfc}\left(\frac{\zeta_{2}}{2\sqrt{\tau }}\right)-e^{a^{2}\tau +a\zeta_{2}}\mathrm{erfc}\left(\frac{\zeta_{2}}{2\sqrt{\tau }}+a\sqrt{\tau }\right)\right],\;\;\;\zeta \leq 0,\;\;\tau >>1.$$

Additionally, accepting $Bi_{r}\to \infty$, solutions (94)–(96) become equal to solutions (90)–(92).

It should be noted that asymptotic solutions (91)–(93) for homogeneous materials of the layer and half-space, taking into account convective cooling on the free surface of the layer, are also new. So far, a suitable solution for maintaining the initial temperature on this surface has been known [37]. This solution is easy to obtain from Formulas (91)–(93), proceeding with them to the limit $Bi_{c}\to \infty$. Then,

(97) $$\Theta^{*}(\zeta ,\tau )≅(1-\zeta )[1-e^{\varepsilon^{-2}\tau }\mathrm{erfc}(\varepsilon^{-1}\sqrt{\tau })],\;0\leq \zeta \leq 1,\;\tau >>1,$$

(98) $$\Theta^{*}(\zeta ,\tau )≅\mathrm{erfc}\left(\frac{\zeta_{2}}{2\sqrt{\tau }}\right)-e^{\varepsilon^{-2}\tau +\varepsilon^{-1}\zeta_{2}}\mathrm{erfc}\left(\frac{\zeta_{2}}{2\sqrt{\tau }}+\varepsilon^{-1}\sqrt{\tau }\right),\;\;\zeta \leq 0,\;\tau >>1.$$

## 3. Results and Discussion

Numerical analysis was carried out in the case of a two-component FGM strip, in which the friction surface was zirconium dioxide ${\mathrm{ZrO}}_{2}$ and the titanium alloy Ti−6Al−4V was chosen as the core. The gray cast iron ChNMKh served as the material of a homogeneous semi-space. The properties of these materials at the initial temperature $T_{0}=20{\;}^{{}^{\circ}}C$, necessary to perform the calculations, were taken from article [47]. The value $\gamma^{*}=1.26$ of the dimensionless gradient of the selected FGM was determined from Formula (1). The remaining dimensionless input parameters were defined by Formulas (9): the spatial variable ζ, the Fourier number τ, and the Biot numbers $Bi_{r}$ and $Bi_{c}$. Numerical integration in the obtained exact solutions was carried out using the QAGI procedure from the QUADPACK package [55]. QAGI is the standard Fortran subroutine for integration over infinite intervals. To launch this procedure, it was necessary to write the real function subprogram definiting the integrand function. Another input parameter was the relative accuracy requested. For our calculations, this parameter was equal to 10^−4^.

The aim of the numerical analysis was to Investigate the mutual impact of dimensionless parameters $Bi_{r}$ and $Bi_{c}$ on the temperature of the friction system. It should be noted that with a fixed value of the Biot number $Bi_{r}$ in the limit case $Bi_{c}\to 0$, the free surface z=d (ζ=1) of the strip was adiabatic (the thermally insulated), and at $Bi_{c}\to \infty$, the initial temperature on this surface was maintained during the entire process of friction heating. On the other hand, with a fixed value of the Biot number $Bi_{c}$, the case $Bi_{r}\to 0$ corresponded to such a heating mode of the considered system, at which the sliding surfaces of the strip and the semi-space were heated separately by heat fluxes with the same intensity equal to the half of the specific power of friction, i.e., $0.5\;q_{0}$. If $Bi_{r}\to \infty$, then the temperature of the friction surfaces of the strip and the semi-space at any time of the heating process should be equal.

**Figure 2:**
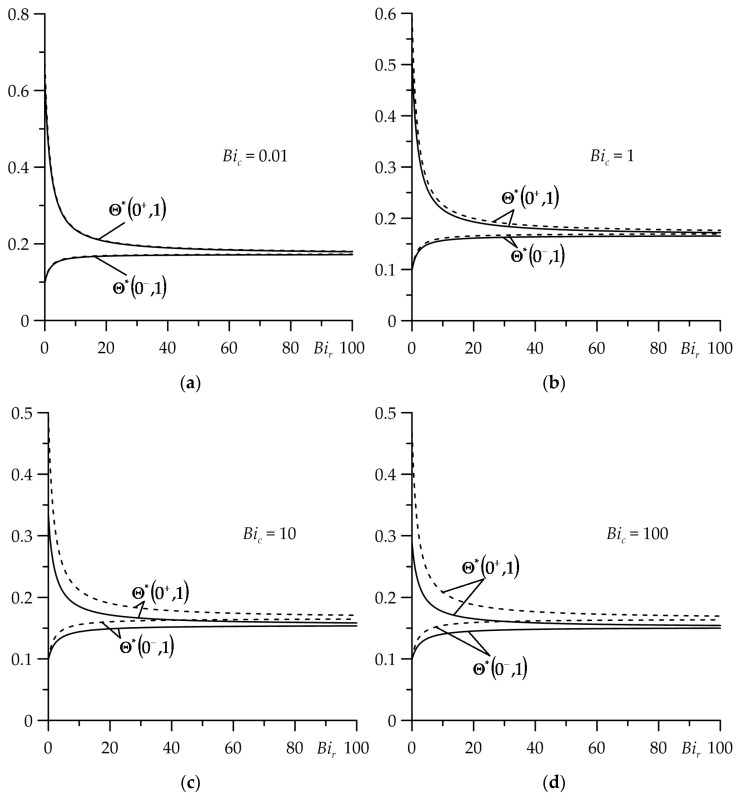
Dependences of dimensionless temperature rise $\Theta^{*}$ on the friction surface of the strip ($\zeta =0^{+}$) and the semi-space ($\zeta =0^{-}$) from the Biot number $Bi_{r}$ for τ=1 at four values of the Biot number $Bi_{c}$: (**a**) 0.01; (**b**) 1; (**c**) 10; (**d**) 100. The continuous curves—the FGM strip; the dashed curves—the strip made of zirconium dioxide.

**Figure 3:**
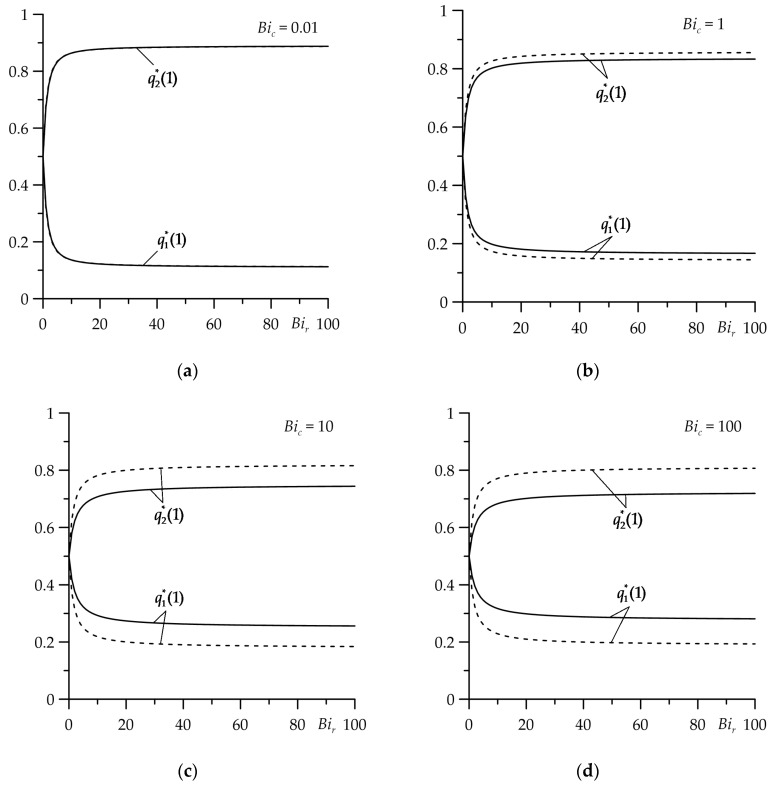
Dependences of dimensionless heat fluxes intensities $q_{i}^{*}$, i=1,2 on the Biot number $Bi_{r}$ for τ=1, at four values of the Biot number $Bi_{c}$: (**a**) 0.01; (**b**) 1; (**c**) 10; (**d**) 100. The continuous curves—the FGM strip, the dashed curves—the strip made of zirconium dioxide.

**Figure 4:**
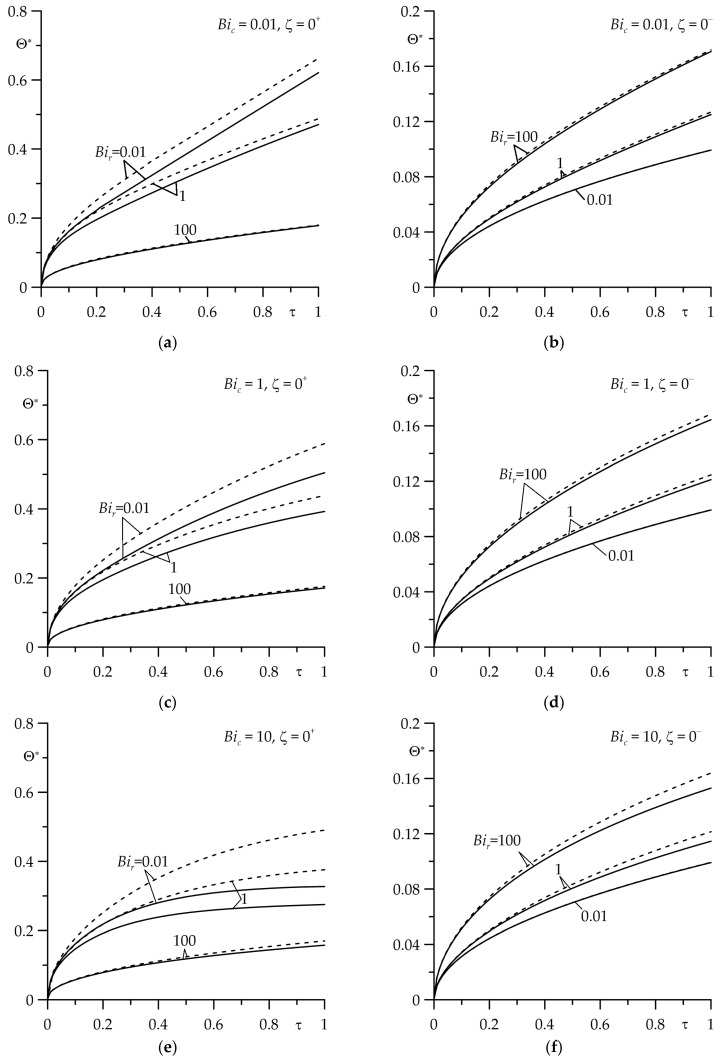

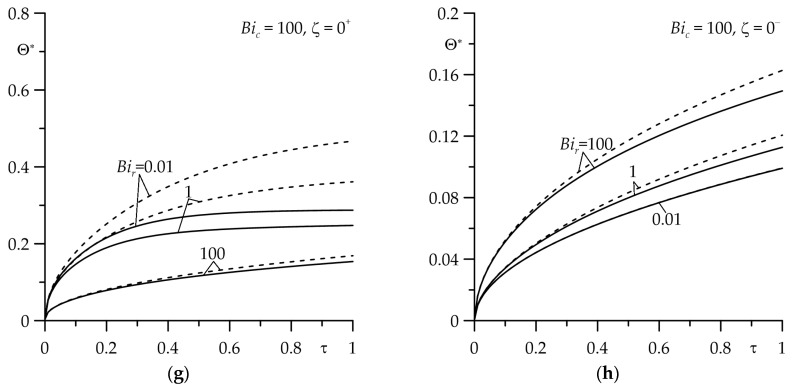
Evolutions of dimensionless temperature rise $\Theta^{*}$ on the friction surface of the strip ($\zeta =0^{+}$) and the semi-space ($\zeta =0^{-}$) for different values of the Biot number $Bi_{r}$ at four values of the Biot number $Bi_{c}$: (**a**,**b**) 0.01; (**c**,**d**) 1; (**e**,**f**) 10; (**g**,**h**) 100. The continuous curves—the FGM strip; the dashed curves—the strip made of zirconium dioxide.

**Figure 5:**
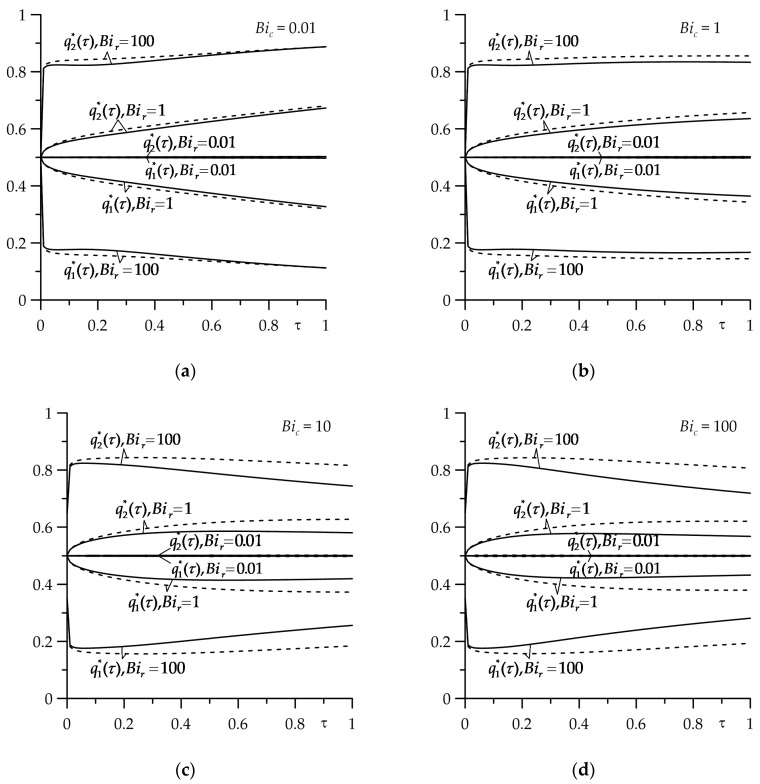
Evolutions of dimensionless intensities of the heat fluxes $q_{i}^{*}$, *i* = 1, 2 for different values of the Biot number *Bi*_r_ at four values of the Biot number *Bi*_c_: (**a**) 0.01; (**b**) 1; (**c**) 10; (**d**) 100. The continuous curves—the FGM strip; the dashed curves—the strip made of zirconium dioxide.

**Figure 6:**
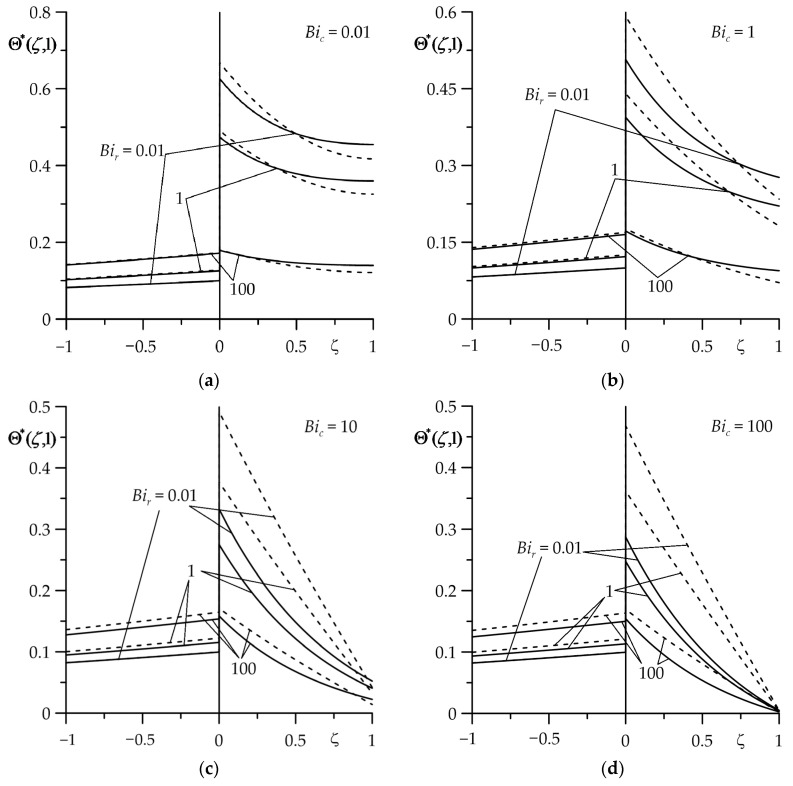
Change in dimensionless temperature rise $\Theta^{*}$ with dimensionless distance ζ from the contact surface ζ=0 at the final moment of time τ=1 of the heating process for selected values of Biot number $Bi_{r}$ at four values of the Biot number $Bi_{c}$: (**a**) 0.01; (**b**) 1; (**c**) 10; (**d**) 100. The continuous curves—the FGM strip; the dashed curves—the strip made of zirconium dioxide.

**Figure 7:**
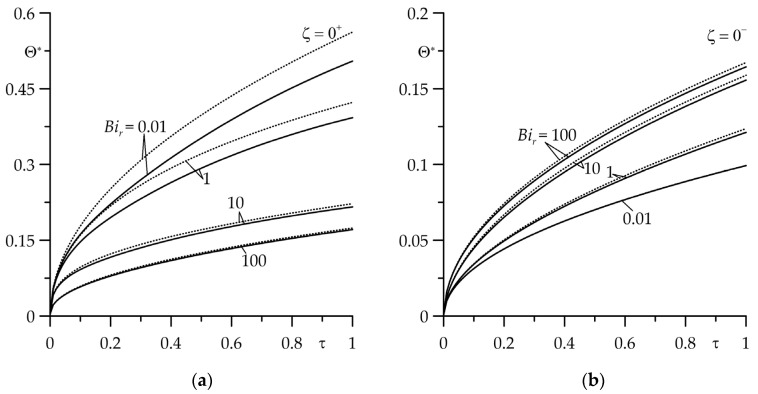
Evolutions of the dimensionless temperature rise $\Theta^{*}$ on the surfaces of friction: (**a**) FGM strip ($\zeta =0^{+}$); (**b**) homogeneous semi-space ($\zeta =0^{-}$) for $Bi_{c}=1$ at four values of the Biot number $Bi_{r}=0.01;\;\;1;\;10;\;100$. The solid curves—the exact solutions (36) and (37); the dotted curves—the asymptotic solutions (80) and (81) at small values of the Fourier number τ.

**Figure 8:**
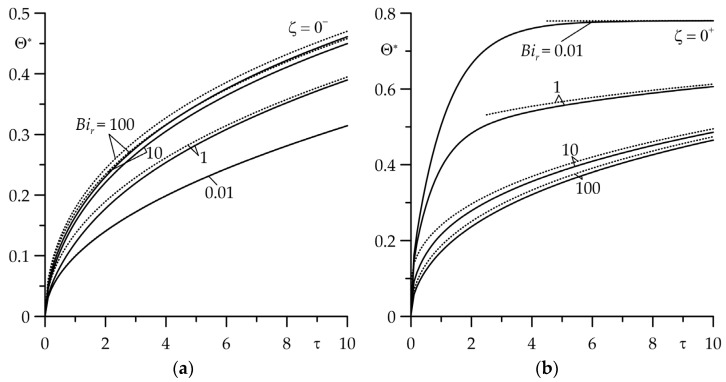
Evolutions of the dimensionless temperature rise $\Theta^{*}$ on the friction surfaces: (**a**) FGM strip ($\zeta =0^{+}$); (**b**) homogeneous semi-space ($\zeta =0^{-}$) for $Bi_{c}=1$ at four values of the Biot number $Bi_{r}=0.01;\;\;1;\;10;\;100$. The solid curves—the exact solutions (29) and (30); the dotted curves—the asymptotic solutions (85) and (86) at large values of the Fourier number τ.

**Figure 9:**
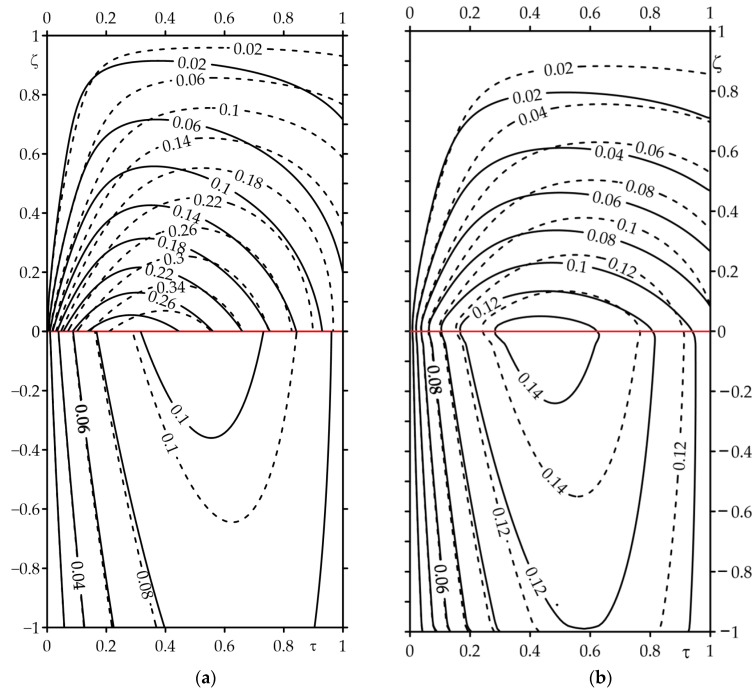
Isolines of the dimensionless temperature rise ${\hat{\Theta }}^{*}\left(\zeta ,\tau \right)$ during braking for $Bi_{c}=100$, $\tau_{s}=1$ at two values of the Biot number $Bi_{r}$: (**a**) 1; (**b**) 100. The continuous curves—the FGM strip; the dashed curves—the strip made of zirconium dioxide.

The results of the calculations are presented in Figure 2, Figure 3, Figure 4, Figure 5, Figure 6, Figure 7, Figure 8 and Figure 9, where the solid curves correspond to the case of an FGM strip (${\mathrm{ZrO}}_{2}/\mathrm{Ti}-6\mathrm{Al}-4V$), and the dashed curves—to the case of the strip made entirely of ${\mathrm{ZrO}}_{2}$. The results, shown by continuous and dashed curves, were obtained using exact solutions, while the dotted curves represent the results obtained on the basis of asymptotic solutions.

The influence of the thermal contact conductivity (the Biot number $Bi_{r}$) on the dimensionless temperature rises $\Theta^{*}$ (39) and (40) of the surfaces of friction of the strip ($\zeta =0^{+}$) and the semi-space ($\zeta =0^{-}$) for the four values of the Biot number $Bi_{c}$ at the fixed value of the Fourier number τ=1 are shown in Figure 2. As expected, the greatest jump in the temperature on the surfaces of friction for both types of strip material took place at small ($Bi_{r}\to 0^{+}$) thermal contact conductivity (high thermal resistance). Increasing the Biot number $Bi_{r}$ equalized the temperature of the friction surfaces of the strip and the semi-space. At $Bi_{r}\approx 100$, their temperature could be considered the same. During the entire heating process, the temperature of the friction system with the FGM strip (the solid curves) was lower compared to the temperature when the homogeneous material of the strip was used (the dashed curves). This effect increased with the growth of the convective cooling intensity (the Biot number $Bi_{c}$) on the free surface ζ=1 of the strip.

The appropriate results for dimensionless intensities of the heat fluxes $q_{i}^{*}$, i=1,2 (54) and (55) are shown in Figure 3. It was noticed that the greatest changes in the intensity of heat fluxes, directed along the normal from the contact surface to the inside of the strip ($q_{1}^{*}$) and the semi-space ($q_{2}^{*}$), occurred in the range $0\leq Bi_{r}\leq 10$. Increasing the thermal contact conductivity (reducing the thermal resistance) resulted in the fact that the amount of heat absorbed by the FGM strip decreased and the heat absorbed by cast iron semi-space increased. With an almost adiabatic process ($Bi_{c}=0.01$), the free surface of the strip, the influence of the gradient of the strip material on $q_{i}^{*}$, i=1,2 was negligible (Figure 3a). With the increase in the intensity of heat transfer (the Biot number $Bi_{c}$) between the free surface of the strip and the surrounding environment, the effect of the FGM of the strip on temperature became more noticeable (Figure 3b–d).

The evolutions of the dimensionless temperature rise $\Theta^{*}$ (39), (40) on the friction surfaces of the strip ($\zeta =0^{+}$) and the semi-space ($\zeta =0^{-}$) for different values of the Biot numbers $Bi_{r}$ and $Bi_{c}$ are shown in Figure 4. It can be seen that with the same input parameters, the use of an FGM strip caused a decrease in the temperature of both elements in comparison to the temperature found in the case in which the strip has a homogeneous material. This tendency is most clearly visible on the friction surface of the strip ($\zeta =0^{+}$) with a small value of thermal contact conductivity ($Bi_{r}=0.01$) and the intensive convective cooling ($Bi_{c}=100$) of the strip. The influence of the gradient nature of the strip material on the temperature on the friction surface of the semi-space ($\zeta =0^{-}$) is much smaller. On this surface, the noticeable temperature differences when using the FGM of the strip and the homogeneous strip material occur in the case of perfect thermal contact of friction ($Bi_{r}=100$) with intensive ($Bi_{c}=100$) convective cooling of the free surface the strip.

Corresponding changes with time (the Fourier number τ) of dimensionless heat flux intensities $q_{i}^{*}$, i=1,2 (54), (55) are illustrated in Figure 5. The much higher thermal conductivity of cast iron ChNMKh compared to ${\mathrm{ZrO}}_{2}$ and Ti−6Al−4V resulted in the fact that the semi-space absorbed most of the heat generated during friction. At $Bi_{r}=0.01$, when the conditions for the separate heating of friction elements were implemented, as expected, the intensities persisted at a constant level of 0.5 during the entire heating process, regardless of the intensity of cooling the exposed strip surface. But already at $Bi_{r}=1$ with the time, the amount of heat directed to the strip monotonically decreased and to the semi-space decreased. The evolutions of the heat flux intensities looked slightly different, with perfect thermal contact of friction ($Bi_{r}=100$). First of all, it was clearly seen that for a fixed value τ the difference between $q_{1}^{*}$ and $q_{2}^{*}$ in this case was the largest compared to the appropriate results obtained at $Bi_{r}=0.01$ or $Bi_{r}=1$. Secondly, the sensitivity $q_{i}^{*}$, i=1,2 to the intensity of the convective cooling on the free surface of the strip increased significantly. If the initial temperature was maintained on this surface ($Bi_{c}=0.01$), a rapid achievement of the minimum value by $q_{1}^{*}$ and the maximum value by $q_{2}^{*}$ was established. Then, with increasing the dimensionless time τ, $q_{1}^{*}$ decreased slightly and $q_{2}^{*}$ accordingly increased. When $Bi_{c}=1$ and the above-mentioned extreme values $q_{i}^{*}$, i=1,2 were reached, further heating practically did not cause their changes. A further increase in the intensity of convective heat transfer on the free surface of the strip ($Bi_{c}=10$, and in particular $Bi_{c}=100$) caused the amount of heat absorbed by the strip to increase after quickly reaching the minimum $q_{1}^{*}$ and maximum $q_{2}^{*}$ values, and that by the semi-space decreased accordingly over the time. The most noticeable such effect was in the case of an FGM strip.

Distributions of the dimensionless temperature rises $\Theta^{*}$ (29) and (30) along the thickness of the strip (0≤ζ≤1) and semi-space (−1≤ζ≤0) at τ=1 for different values of the Biot numbers $Bi_{r}$ and $Bi_{c}$ are presented in Figure 6. At a fixed-value $Bi_{c}$ in the case of $Bi_{r}=0.01$, the temperature jump was clearly visible on the contact surface ζ=0. At $Bi_{r}=1$ this jump decreased, and at $Bi_{r}=100$ it became practically imperceptible. Comparing to the case of a homogeneous strip, the FGM strip did not show a significant impact on the temperature of the semi-space. It could be observed that the temperature of the semi-space decreased linearly with distance from the contact surface, regardless of the choice of strip material. A different, non-linear nature of the temperature distribution was observed in the strip. At the same time, the use of FGM reduced the temperature of the strip in comparison to the temperature of the strip made of zirconium dioxide. This effect was most noticeable with intensive ($Bi_{c}=10$ or $Bi_{c}=100$) cooling on the free surface (ζ=1) of the strip.

The next part of the numerical analysis was devoted to determining the time intervals in which the asymptotic solutions could be used to estimate the temperature of the friction surfaces of the FGM strip ($\zeta =0^{+}$) and the homogeneous semi-space ($\zeta =0^{-}$) at small (73), (74) (Figure 7) and large (85), (86) (Figure 8) values of the Fourier number τ (9). The temperature values obtained by these solutions (the dotted curves) were compared with the corresponding results found using the exact solutions (29), (30) (the solid curves). It was established that the usefulness of the asymptotic solution (73) to determine the temperature of the friction surface of the FGM strip largely depended on the value of the Biot number $Bi_{r}$: results obtained from the exact (29) and asymptotic (73) solutions, which differed slightly for $Bi_{r}=0.01$ or $Bi_{r}=1$ at 0≤τ≤0.1 and for $Bi_{r}=10$ or $Bi_{r}=100$—if 0≤τ≤1 (Figure 7a). On the other hand, an asymptotic solution (74) for the semi-space could be used to estimate the temperature of the surface of friction at 0≤τ≤1 for four selected values of $Bi_{r}$ (Figure 7b).

Similar sensitivity to the value of the Biot number *Bi_r_* of the results obtained by means of an asymptotic solution (85) for the FGM strip at high values τ is visible in Figure 8a. The difference in the results obtained by means of exact (30) and asymptotic (86) solutions for $Bi_{r}=0.01;\;\;1$ was insignificant at τ≥5, and for $Bi_{r}=10;\;100$, it was permissible not only at large but also at small values τ. The asymptotic solution (86) for semi-space at high values of the Fourier number could be used at τ≥0 (Figure 8b).

Isolines of the dimensionless temperature rise ${\hat{\Theta }}^{*}\left(\zeta ,\tau \right)$ (47)–(49) during single braking with constant deceleration with the forced (*Bi_c_ =* 100) convective cooling of the free surface of the strip for two values of the Biot number *Bi_r_* is demonstrated in Figure 9. First of all, it should be noted that the temperature of the strip made of FGM was lower compared to the case of applying a layer of zirconium dioxide to the material. Secondly, just like during sliding with constant specific friction power, the jumps of isolines were also visible when passing through the contact surface in the case of *Bi_r_ =* 1 (Figure 9a). However, for *Bi_r_ =* 100, the temperature of the friction surfaces of the strip and semi-space were the same (Figure 9b). At low contact thermal conductivity (*Bi_r_ =* 1), the temperature of the strip was higher than the semi-space temperature (Figure 9a). The increase in parameter *Bi_r_* equalized the temperature of both elements of the friction pair (Figure 9b). The effect of the linearly decreasing time profile of the specific friction power was visible primarily in the achievement of the maximum temperature of both elements at a fixed distance from the contact surface, not at the stop moment $\tau =\tau_{s}=1$ (as it was in the case of sliding with constant specific friction power), but within the time interval $0<\tau <\tau_{s}$.

## 4. Conclusions

A mathematical model to determine the temperature field resulting from heating during the sliding of a strip made of FGM on a homogeneous semi-space surface was developed. The influence of two dimensionless input parameters, namely the Biot numbers *Bi_r_* and *Bi_c_*, defined by Formula (9), on the temperature of such a friction system was examined. The first of them (*Bi_r_*) was directly proportional to the thermal contact conductivity *h_r_*, which in turn was inversely proportional to the thermal resistance of the friction surface of the strip and semi-space. Thus, at a fixed value of the contact pressure, the higher the roughness of these surfaces, the greater their thermal resistance and the lower the thermal contact conductivity.

Consideration of thermal contact conductivity in the modeling of the frictional heat generation process was carried out by means of two boundary conditions on the contact surface. The first of them assumed that the sum of the intensities of heat fluxes absorbed on the contact surface along the normal to this surfaceinside each element of the friction pair was equal to the specific power of friction, i.e., the product of the coefficient of friction, the contact pressure, and the sliding speed. The second condition stated that the difference in the above-mentioned intensity of heat fluxes was proportional to the difference in the temperature of friction surfaces. The ratio of proportionality here was the thermal contact conductivity *h_r_* or in the dimensionless form of the Biot number *Bi_r_*. Both of these conditions created the so-called conditions of imperfect thermal contact of friction. A characteristic of the solutions of appropriate heat problems of friction, obtained under such boundary conditions, is the temperature jump on the contact surfaces of the sliding bodies. It should be noted that during the sliding of smooth surfaces, the thermal resistance became negligible and the value of the parameter *Bi_r_* was large. In the limit case, $Bi_{r}\to \infty$, the temperature difference in the friction surfaces disappeared. So, it can be said the generation of heat takes place in conditions of perfect thermal friction contact.

The Biot number *Bi_c_* characterized the intensity of convective cooling on the exposed surface of the strip by means of the heat transfer coefficient *h_c_*. The influence of the parameter *Bi_c_* on the temperature of the system operating in conditions of perfect thermal contact of friction was examined in article [48].

Numerical analysis was performed for the FGM (${\mathrm{ZrO}}_{2}/\mathrm{Ti}-6\mathrm{Al}-4V$) strip and homogeneous (gray cast iron ChNMKh) semi-space. At the same time, the temperature of the system, consisting of a homogeneous (${\mathrm{ZrO}}_{2}$) strip and a cast iron (ChNMKh) semi-space, was analyzed. It was established that:

1. Regardless of the value *Bi_c_*, the perfect thermal contact of friction occurs when *Bi_r_ ≅* 100.
2. The use of the functionally graded material on a strip reduces its temperature compared to the case of homogeneous material. The greater the decrease in temperature, the smaller the parameter *Bi_r_* and the higher the parameter *Bi_c_* is. The effect of the gradient nature of the strip material on lowering the temperature of the semi-space is insignificant.
3. Most of the heat generated during friction is absorbed by the cast iron semi-space. With a fixed value of the Biot number *Bi_c_*, the greatest changes in the intensity of heat fluxes, directed along the normal to the contact surface between the strip and the semi-space, occur in the range of 0 ≤ *Bi_r_* ≤ 10. As the parameter *Bi_c_* increases, the impact of the gradient of the strip material on the intensity of heat fluxes becomes more noticeable.
4. The obtained asymptotic solutions at small and large values of the Fourier number *τ* can be used with sufficient accuracies to estimate the temperature of the considered friction pair. Increasing the parameter *Bi_r_* causes the widening of the interval of the parameter *τ*, in which the temperature of the strip obtained from asymptotic solutions slightly differs from the temperature determined by the exact solution. Such an effect was not noticed when determining the temperature on the friction surface of the semi-space: the appropriate asymptotic solutions allow one to obtain satisfactory results in the entire range of the Fourier number changes.
5. With a linearly decreasing time profile of the specific friction power, the spatial–temporal temperature distribution in the strip and semi-space is non-uniform. At each set distance from the contact surface, the temperature reaches its maximum value within the heating time interval. In a strip made of FGM, the moment of reaching the maximum temperature approaches the stop moment as the distance from the contact surface increases. However, when using a homogeneous layer and half-space materials, the maximum temperature is reached in approximately half the heating time.

In the future, the authors intend to develop a mathematical model to determine the temperature of the friction system of two FGM strips with imperfect thermal contact of friction and convective heat exchange with the surrounding environment on their free surfaces.

## Data Availability

Data are contained within the article.

## Nomenclature

$Bi_{c}$	Biot number on the free surface of the strip
$Bi_{r}$	Biot number through the contact surface
c	Specific heat ($J\;{\mathrm{kg}}^{-1}K^{-1}$)
d	Thickness of the strip (m)
$h_{c}$	Coefficient of convective heat exchange on the free surface of the strip
$h_{r}$	Coefficient of contact heat conductivity through the surfaces
$I_{n}(\cdot )$	Modified Bessel functions of the *n*th order of the first kind
$J_{n}(\cdot )$	Bessel functions of the *n*th order of the first kind
$K_{n}(\cdot )$	Modified Bessel functions of the *n*th order of the second kind
k	Thermal diffusivity ($m^{2}s^{-1}$)
K	Thermal conductivity ($W\;m^{-1}K^{-1}$)
q	Specific power of friction ($W\;m^{-2}$)
$q_{0}$	Nominal value of the specific friction power ($W\;m^{-2}$)
t	Time (s)
$t_{s}$	Stop moment of the process (s)
T	Temperature (${}^{{}^{\circ}}C$)
$T_{0}$	Initial temperature (${}^{{}^{\circ}}C$)
$Y_{n}(\cdot )$	Bessel functions of the *n*th order of the second kind
z	Spatial coordinate in axial direction (m)
$\gamma^{*}$	Gradient parameter of FGM
Λ	Temperature rise scaling factor (${}^{{}^{\circ}}C$)
ε	Dimensionless coefficient of thermal activity
Θ	Temperature rise (${}^{{}^{\circ}}C$)
$\Theta^{*}$	Dimensionless temperature rise
${\hat{\Theta }}^{*}$	Dimensionless temperature rise during sliding
ρ	Density ($\mathrm{kg}\;m^{-3}$)
τ	Dimensionless time
$\tau_{s}$	Dimensionless stop time
ζ	Dimensionless spatial coordinate in axial direction
